# Delineation of endorheic drainage basins in the MERIT-Plus dataset for 5 and 15 minute upscaled river networks

**DOI:** 10.1038/s41597-023-02875-9

**Published:** 2024-01-10

**Authors:** Alexander A. Prusevich, Richard B. Lammers, Stanley J. Glidden

**Affiliations:** Institute for the Study of Earth, Oceans, and Space, University of New Hampshire, Durham, NH 03824 USA

**Keywords:** Hydrology, Environmental impact

## Abstract

The MERIT-Hydro networks re-gridded by the Iterative Hydrography Upscaling (IHU) algorithm do not retain exo- or endorheic basin attributes from the original data. Here we developed methods to assign such attributes to those and any other digital river networks. The motivation is that endorheic inland drainage basins are essential for hydrologic modelling of global and regional water balances, land surface water storage, gravity anomalies, sea level rise, etc. First, we create basin attributes that explicitly label endorheic and exorheic catchments by the criteria of direct or hidden connectivity to the ocean without changing their flow direction grid. In the second step we alter the delineation of endorheic basins by the merging algorithm that eliminates small inland watersheds to the adjacent host basins. The resulting datasets have a significantly reduced number of endorheic basins while preserving the total land portion and topology of the inland basins. The data was validated using the Water Balance Model by comparing volume of endorheic inland depressions with modelled water accumulation in their inland lakes.

## Background & Summary

While connectivity of surface water flows, such as streams and rivers, determine individual river basins or catchments, their drainage to the ocean or to inland seas and depressions classifies them as exorheic or endorheic basins respectively^1^. Unlike in natural environments where that definition of river catchments can be an oversimplification^2^, in this work we use a straightforward definition of basin types as those are directly derived from the digital river networks in almost all hydrological computer models.

Digital river networks with flow direction data^3–9^ are essential for hydrological^10–13^, ecosystem^14,15^, water resource management^10,16,17^, hydro-infrastructure^18–20^, and other geoscience models especially in those that simulate surface water routing in streams and rivers^21^. Flow direction data defines other important entities of the hydrological systems such as river stream order, tributaries, and the extent of the entire river basin. Each drainage basin has an outlet point or river mouth representing the last downstream grid cell of the directional tree graph that defines the river network. Exorheic river basins drain water to the world’s oceans, and, alternatively, there are endorheic (internal) basins that terminate on land and whose river mouths do not connect to the ocean. These endorheic basins terminate at inland lakes or dry depressions. All endorheic basins comprise about 20% of the global land area^1^. Modelling water storage in these endorheic lakes is very important for understanding its historical dynamics^22,23^, flooding or recession mitigation^24,25^. Additionally, interpretation of total water storage (TWS) contributions to the gravity anomalies recorded by the GRACE satellite is important^26–28^.

In many hydrological models, the river network is stored in a two-dimensional matrix where each cell specifies the direction of downstream flow from that grid cell to the next downstream cell. Being a special case of graph theory^29,30^, namely a directional tree graph, the flow direction matrix records the direction of neighbouring cells of the regular grids by a bit value in a byte number allowing a byte to record all 9 possible directions for flows into and out of a grid cell. Each of the 8 bits in a byte have a Boolean meaning for all 8 cardinal directions starting from east and continuing clockwise^31^. Eight zeroes in the byte indicate “no flow” or a river mouth outflow condition. Unfortunately, there is no additional room in the 8-bit byte value to hold an extra bit combination to indicate one of the two river mouth flow types, exorheic or endorheic. In some special cases, the network authors sacrifice the limitation of strict flow-in to/from flow-out conversion to record flow-out exorheic mouth direction as byte zero and endorheic mouth direction as byte −1 which are the wrap-around values of 255^5^. Using this work-around solution may help to differentiate the river mouth types in flow-out direction grids where only one outflow direction is allowed, but it requires off-splitting the exorheic mouth Boolean grid and patching back zero values to the outflow direction grid. The latter likely have been implemented in some hydrological packages, and we have implemented this in the University of New Hampshire Water Balance Model (WBM^32^).

The recently published high resolution MERIT river network dataset^5^ does include the special value −1 (255 as a wrap-around byte value), but more recent upscaled derivatives of this product at coarser resolutions by Eilander and others^6^ did not retain this information making these upscaled networks difficult to use in hydrological models where endorheic basins are explicitly modelled.

In exploring solutions to this oversight, we found that a simple solution to determine endo- and exorheism of the basins was not possible from just the flow direction. Specifically, the absence of nodata cells among the basin mouth neighbours indicate an endorheic basin since a nodata value is assumed to be non-land, and, thus, represents ocean grid cells. This seemingly simple method does not work as many near coastal river mouth cells may have narrow passages, such as estuaries or fiords, connecting them from inland to the ocean which exist on the sub-grid cell level but are not reflected in the coarser resolution upscaled flow direction grid.

This paper describes a river mouth delineation method to determine whether or not a drainage basin is endorheic or exorheic in the Eilander data^6^ upscaled river network datasets. In addition, we use optional methods for removal of numerous small endorheic watersheds. We tested and validated those methods over the upscaled 5 and 15 arc minute MERIT networks and the resulting value added datasets, with the additional basin type information, are available in the data repository^33^.

The MERIT-Plus river network datasets (in 5 and 15 arc minute resolution) discussed here add value to the original upscaled IHU MERIT data. The main purpose of this work to identify the endorheic and exorheic basin types which are missing in the source datasets. Merging (cleanup) of small endorheic basins introduced a few local changes in flow direction and basin ID data, but made the datasets more suitable for a broader range of hydrological modelling applications that simulate water balance and accumulation in the endorheic lakes and land depressions. Those applications are relevant to studies of climate change impact, the hydrological cycle in arid areas, interpretation of historical and seasonal gravity anomaly trends, water resource management, ecosystem protection, and endorheic lake assessment.

## Methods

### Source data

**Fig. 1:**
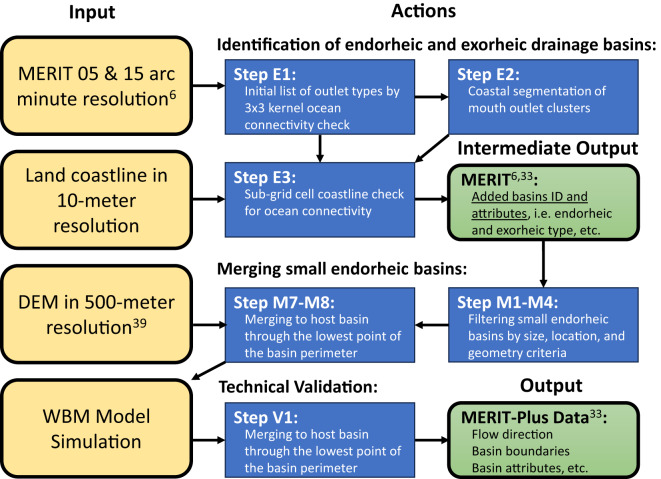
Flowchart of the workflow to produce the MERIT-Plus datasets. Left column objects are the processing inputs (tan); middle and right column objects are the processing actions (dark blue) and outputs (green).

To develop and test basin type delineation methods we used the 5 and 15 arc minute networks produced by the Iterative Hydrography Upscaling (IHU) method^6^. These were upscaled from the original 3 arc second MERIT Hydro raster-based river network by Yamazaki and others^5^. The IHU upscaled datasets do not retain the river mouth type (endorheic or exorheic) from the source data. The IHU method description does not indicate a reason for this, and we assume the IHU method uses only flow direction data and no additional information to resolve this issue. The methods described below can be contributed to the future versions of the IHU open-source software for its additional utility to delineate drainage basin type (Fig. 1).

In a flow direction dataset all grid cells over land have a valid flow direction value, and therefore a nodata grid cell can be considered as part of the ocean. Identifying exorheic and endorheic basins cannot be carried out through a simple check of adjacency between the basin mouth grid cell and a nodata cell (exorheic) or a basin mouth grid cell with no adjacent nodata cell (endorheic). Unfortunately, this simple approach does not work due to several limitations:

1. there can be a sub-grid cell level narrow passage to the ocean such as a fiord, estuary or human-made channel that is not reflected in a coarser scale grid;
2. unresolved low-land or shallow ocean delta areas covered with vegetation (e.g., mangrove forests in tropics and ice fields in Arctic zones) causing those areas to be seemingly disconnected from the ocean;
3. grid cells over water bodies of large rivers (e.g., Amazon, Lena, Indus) where river channel width exceeds cell size so that it has undesignated flow direction that gives a false single cell drainage basin; and
4. digital elevation model (DEM) data or other errors in the digital river network algorithm that lead to false local flow directions.

Identification of basin types here does not change river flow direction data and basin boundaries. The primary goal of this task is to add endorheic/exorheic basin type labels to all basins that had been lost in the upscaled river network products as described in the “Source Data” section above.

If only the adjacency check is used for the basin type identification in the MERIT-Hydro IHU 6, then most land area would be wrongly classified as endorheic since limitation #3 above will falsely mark all major river basins as endorheic. In order to resolve river mouth connectivity to the ocean and, thus, river basin type attribution, we present here a multi-step procedure to both identify basin type and to merge many smaller endorheic basins into larger drainage basins.

**Fig. 2:**
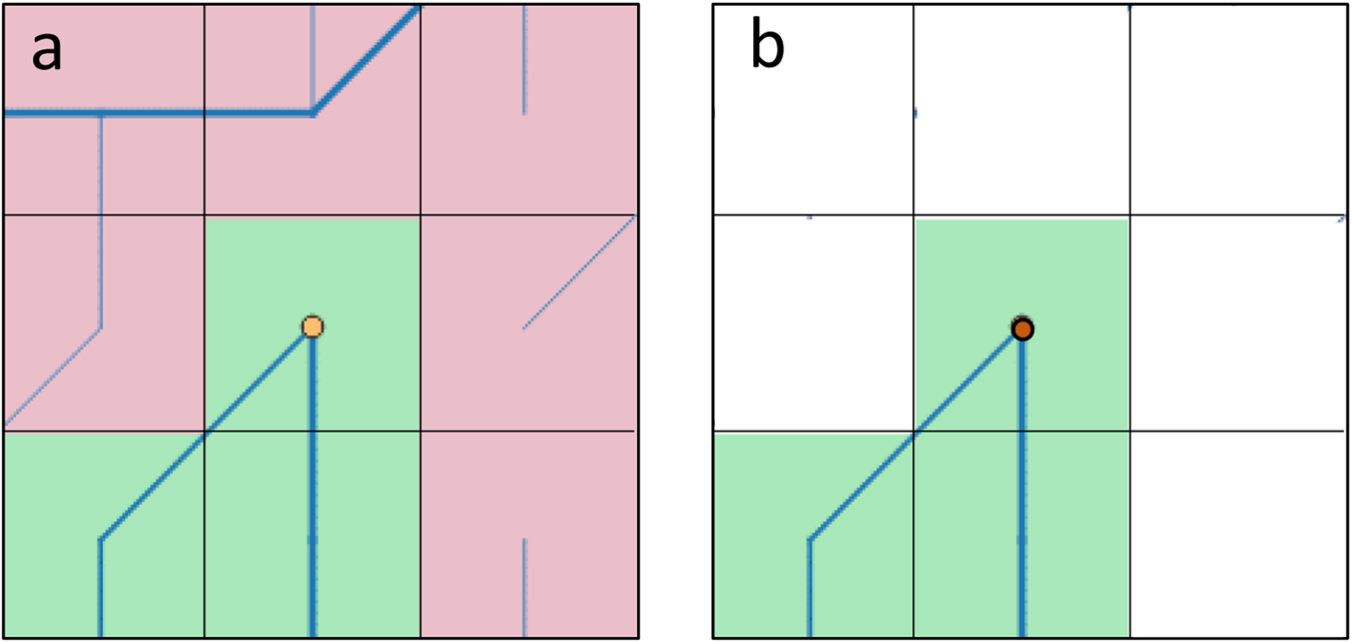
Determining potentially endorheic drainage basin mouth grid cells by searching for nodata cells in the 3 × 3 kernel around each river mouth cell: (**a**) potentially endorheic basin (green) with mouth point indicated with a yellow circle as it is surrounded by other land grid cells (light red) yielding 9 valid cells count in the kernel; (**b**) exorheic outlet of a basin (green) shown bordering 6 nodata grid cells (white), with the number of land cells being 3 in this example. Blue lines indicate cell connectivity from the flow direction data.

**Step 1** - Create initial list of all mouth outlets and assign first classification:

This is done by filtering out all outlets and checking them for the absence of nodata values in the adjacent grid cells (Fig. 2). This results in a list of exorheic mouth points, and the list of potentially endorheic outlets to be further processed in subsequent steps.

This is implemented through a sequence of straight forward actions:

1. Filtering all river mouth grid cells by flow direction value equal to zero. The result is a list of locations (longitude/latitude and/or column/row) of all mouth points.
2. Identification of exorheic mouth points by searching a 3 × 3 kernel around each mouth point and checking for the count of non-nodata cells (i.e., ocean). If the count of non-nodata cells in the kernel is 9, meaning that the river mouth point is surrounded by land cells, then it is listed as a potentially endorheic outlet. A kernel count less than 9 indicates the presence of nodata cells next to the mouth point making it a coastal cell and therefore an exorheic outlet.
3. Lists of both types of outlets resulting from this step are also transferred to binary mask layers for use in the next segmentation step.

**Fig. 3:**
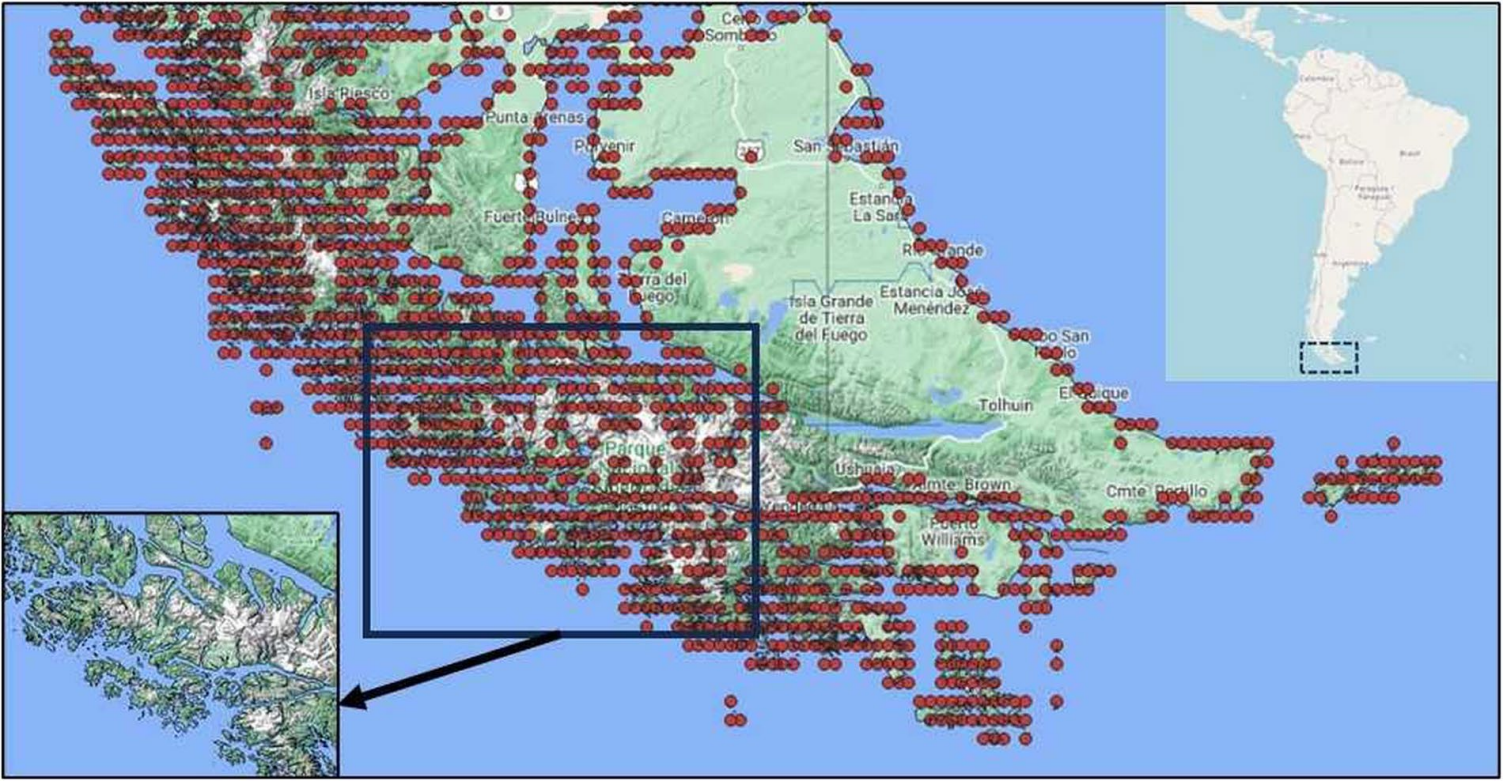
Identification of endorheic mouth grid cells by coastal segmentation of mouth point clusters. Shown here is the large fiord region along the southern coast of South America spanning Chile and Argentina near Cape Horn. Index map is in the upper right corner, and the view of ocean inlet land is in the lower left corner indicating that the cluster or basin outlets is indeed connected to the ocean.

**Step 2** - Coastal segmentation of mouth outlet clusters:

Both, the original 3 arc second MERIT network and the set of upscaled MERIT river network datasets allow a grid cell to have a flow direction value if it is part land and part ocean. Entirely freshwater grid cells in the estuary of large rivers, such as the Amazon, also have zero values for the flow direction making it potentially endorheic in Step 1. However, these “mouth” grid cells do not accumulate water over time as endorheic lakes and, therefore, cannot be included as endorheic basins. In this step we change the classification of these false endorheic outlets by checking whether these cells are adjacent to a coastal exorheic outlet grid cell or can be connected to it through the chain of other such false outlets (Fig. 3). If a potentially endorheic outlet (i.e., all adjacent cells have no nodata values) can be connected to the coast through a continuous chain of other exorheic outlets, then it is identified as exorheic. The rationale of this step is checking adjacency and/or chain adjacency conditions which does not require the use of any additional dataset, such as a high-resolution ocean coastline vector or ocean high resolution grid mask, making this step self-sufficient and solely based on the flow direction source data itself.

Software implementation of this step is done utilizing an Image2D package that is coded in many popular computer languages. We use the Perl PDL implementation in this work^34^. Its function “cc8compt()” performs image segmentation by labelling spatially continuous areas (clusters) where values are non-zero, thus providing a mask of river outlet points on a grid of both types. The number eight in the function name indicates that connectivity is checked through each of the four pixel sides and four corners (8-connected cells). Workflow here has two actions:

1. Perform segmentation of the gridded mask layer of all outlet points, which results in a map of labelled clusters of continuous outlet pixels.
2. Each cluster is then checked for intersections with coastline pixels (i.e., pixels bordering nodata grid cells). If there is an intersection with a coastline, then all outlets in this cluster are identified as exorheic.

This step works on the assumption that there are sub-grid cell level passages (such as fiords) to the ocean, and, thus, there cannot reasonably be an endorheic outlet next to the exorheic mouth cell. A close visual inspection of many clusters confirms a presence of sub-pixel passages to the ocean for each grid cell in the cluster (Fig. 3). We used an ultra-high resolution (10 m) land and coastline vector dataset^35^ to make sure that all chained coastal cluster grid cells are connected to the ocean.

**Fig. 4:**
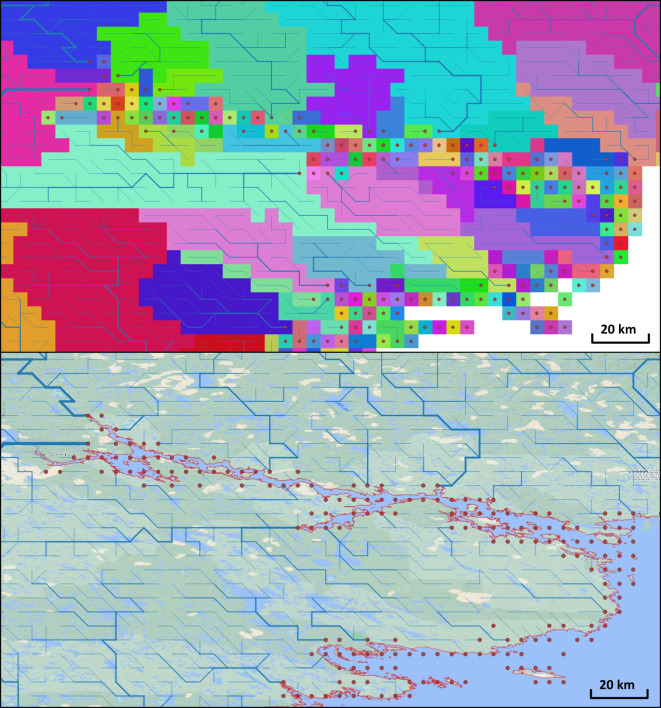
Identification of inland but connected to ocean exorheic basins. Top panel – Basins (coloured polygons) and their river mouth points (red circles) that are do not appear to be connected to the ocean (blue) ion the original gridded field dataset. Bottom panel - High resolution (10 m) ocean coastline vector dataset^35^ (thin red lines) is used to check mouth outlets that are connected to the ocean in their sub-grid cell level either directly or within the user-specified distance. In this example, located in region of (Chesterfield Inlet, of Hudson Bay, in Nunavut, Canada,) a distance of 20 km is used, and all basin outlet cells (red dots) are classified as exorheic.

**Step 3 (optional)** - Use an ocean or land mask:

In this step, we apply a high-resolution vector or grid land mask to check whether the mouth outlet grid cell contains an ocean or located at a minimal distance from the ocean (Fig. 4). The minimal distance is an input parameter with default values set to zero which means no checking by distance. This step is optional as it involves the use of an additional high-resolution land mask dataset which may not be available to the user. In this work we used land vector polygons at 30 m segments from OpenStreetMaps (https://www.openstreetmap.org).

### Merging small endorheic drainage basins with neighbouring drainage basins

Identification of endorheic and exorheic drainage basins described in the previous section allows for the classification of many river basins to the exorheic and endorheic types based on the location of river mouth points relative to their location or their cluster location of nodata values grid cells, and the proximity to a high-resolution ocean coastline. No flow direction itself has been altered, however, close visual inspection of the remainder of endorheic basins and their mouth points in the upscaled MERIT river network indicates that there are a large number of small endorheic basins that may need to be removed from the network by merging them to adjacent watersheds. This process of merging basins necessitates a change in flow direction of the network and basin ID mask in those areas where merging is performed.

Whether or not a drainage basin flows to the ocean is a function of its topography, geomorphology, and water balance. Because endorheic basins are partly defined by their climate, any hydrological model run at century time scales will have variation in the water balance and small basins with low “pour points” that are currently endorheic could reconnect to a larger adjoining basin. Also, small size (e.g., up to 100 km^2^) endorheic basins inside a larger basin requires further assessment, especially if its mouth point is located at its own basin boundary. The location of an outlet point at its own basin boundary of a small endorheic basin can be the result of unresolved connectivity through a narrow canyon-like passage that is not reflected in the source DEM dataset used to produce the river flow data. We therefore implemented a routine to identify those small basins and to reconnect them to their larger adjacent basins. This had the effect of reducing the total number of endorheic drainage basins across the globe while leaving the total endorheic area in the IHU data (after performing steps # 1–3 above) with little change.

**Fig. 5:**
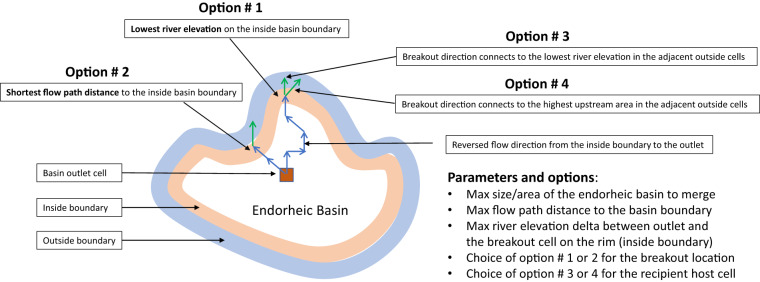
Merging small endorheic basins to an adjacent host watershed: approach and the list of parameters and options.

We have employed a multi-option approach to provide flexibility in which endorheic basins are merged. This is illustrated in Fig. 5 in which the following sequence of steps is implemented to process each endorheic basin:

1. Filter out all endorheic basins by maximum drainage basin area size parameter. This is the most essential filter since we target for merging only small basins such that their removal will not significantly change the total global endorheic land area. Most of these small basins merge into a host basin which is also endorheic. These cases do not change the total endorheic area.
2. Locate all inside and outside basin boundary grid cells and record the elevation of each. The MERIT dataset comes with the minimum river surface elevation in a grid cell which we use here and refer as “elevation”.
3. For each inside boundary cell, trace the flow path to the basin outlet cell, record it and its flow path length.
4. Identify and mark the pour point grid cell on the inside boundary by the lowest elevation (user option #1, Table 1) or minimum flow path length (user option #2) criteria. Note, these options are mutually exclusive and cannot both be set to True or False, and search for the lowest elevation pour point is skipped if option #2 is set to False.

**Table 1:** Parameters and options from endorheic merging numerical procedure.

ID*	Type	Description	Default value
P1	Parameter	Maximum area (km^2^) of the endorheic basin.	10^4^ km^2^
P2	Parameter	Maximum elevation difference (m) between the basin outlet and pour point cells.	100 m
P3	Parameter	Maximum flow path length (km) between endorheic basin outlet and pour point cell of the basin. 0 km distance means that the basin outlet cell must be on the basin boundary.	0 km
Op1	Option: Pour point location	Use lowest elevation of the endorheic basin boundary/perimeter. We used the NASA 500-m DEM^40^	False
Op2		Use the shortest flow path distance from the outlet to the pour point cell. Automatic negation of the option #1.	True
Op3	Option: Connecting the pour point cell to the adjacent host basin	Use lowest elevation of the adjacent basin cells that are adjacent to the endorheic basin pour point cell	False
Op4		Use the highest upstream area of the adjacent basin cells that are adjacent to the endorheic basin pour point cell. Automatic negation of the option #3.	True

*P1, P2, and P3 are required parameters and the rest are optional.
5. Check whether the difference in elevation between the pour point and outlet cell (if option #1 is set to True) or the flow path length is equal to or less than the corresponding “Maximum flow path length” input parameter value (if option #2 is set to True). Skip this basin or continue to the next action items based on this check outcome.
6. Trace the flow path from the pour point cell to the outlet and reverse the flow direction of each of the grid cells along that path (Fig. 5).
7. Connect the endorheic basin to the adjacent watershed using the chosen user input option #3 or #4 (lowest elevation or highest catchment area respectively) in the outside boundary cells that are directly adjacent to the pour point. This is done by setting the flow direction on the pour point grid cell toward a cell in one of the adjacent basin’s cells that meet the chosen criteria. Note, these options are mutually exclusive and cannot both be set to True or False. Also, the lowest elevation of the grid cell is not necessarily the same as the highest catchment area cell, because the elevation dataset represents the average grid cell elevation while the river path is near the minimum elevation of that cell and the difference between cell average and minimum elevation can be significant.
8. Set all basin ID grid cells of the merged watershed to the basin ID of the host watershed it is merged to.

The available parameters and options for endorheic basin identification and merging procedure are listed in Tables 1, 2. If only the adjacency check is used for the basin type identification in the MERIT-Hydro IHU 6, then most land area would be wrongly classified as endorheic since limitation #3 above will falsely mark all major river basins as endorheic.

**Table 2:** Parameters and their values used for MERIT-Plus data production in 5 arc minute resolution.

Parameter	Value	Comment
Identification		
Coastal segmentation	True	
Ocean mask	Mask file	Data from OpenStreetMaps
Ocean coast buffer	25 km	About double cell size
Merging (see Table 1)		
Max area (P1)	2500 km^2^	Visual equivalent of 50 × 50 km square
Max elev. delta (P2)	100 m	
Max flow path (P3)	0 km	Outlet is on the endorheic basin boundary
Breakout (Op1 & 2)	Op1 = False	The shortest distance from outlet to boundary
	Op2 = True	
Connecting (Op3 & 4)	Op3 = False	The highest upstream area in host basin cells adjacent to the breakout cell
	Op4 = True	

### Auxiliary datasets

These methods were applied to upscaled 5 and 15 arc minute MERIT datasets for the endorheic basin identification and elimination of outliers that met certain criteria. These were flow direction and river elevation data sets. Upstream area and the basin ID mask were derived from flow direction data. Basin attributes were also derived from the UNH river database^7,32^ by matching each MERIT basin’s spatial extent to the known named rivers. Additional attribute files included the names of the host continent, receiving ocean, sea basin, and other characteristics such as basin area and main river length.

Another auxiliary dataset that we used for basin type identification and which does not belong to the MERIT package, is the high resolution land vector polygons from the OpenStreetMaps. The use of this dataset or any other for land/ocean masking is optional, but it helps to resolve connectivity of some river outlets to the ocean, especially, over Arctic and sub-Arctic lands where land elevation gradients are very low allowing very narrow ocean water passages to propagate far inland, but only at the sub-grid cell level only.

### Products

We refer to the products of this work as MERIT-Plus reflecting value-added rationale in the delineation of the endorheic basins. As it is described in the previous sections, production of MERIT-Plus datasets involves two fundamental procedures: (1) identification that does not alter the original river flow direction, and (2) merging of small endorheic basins matching certain criteria to their adjacent host basins where this procedure changes the original flow direction and basin ID data. The resulting identity of the endorheic basins is recorded by two added specialized data layers:

1. Gridded layer for endorheic basin IDs only where all other grid cells (ocean and exorheic basins) have nodata values.
2. Signed integer data type for flow direction where endorheic outlets have a conventional value of −1. The numerical value for the flow direction of exorheic outlets is zero.

We used the open source GDAL driver AAIGrid (Arc/Info ASCII Grid) format for these layers which, if needed, can be readily converted to any other GDAL supported format (e.g., GeoTIFF, netCDF) by user preference (http://www.gdal.org/).

## Data Records

The MERIT-Plus data public access, use, re-use, and re-distribution is warranted and compliant with the terms and conditions for data sharing by the “International Creative Commons Attribution 4.0” license.

Two upscaled 5 and 15 min MERIT source datasets^6^ have been processed for MERIT-Plus products. Important parameters, statistics, and basic validation for each of those spatial resolutions are discussed in the sections below as well as in the “README MERIT-Plus Dataset-v2.2.pdf” file of the MSD-LIVE repository^33^.

**MERIT-Plus 05 minute v2.2 Data:**

**Repository**: MSD-LIVE^33^, Project: Program on Coupled Human and Earth Systems (PCHES) https://data.msdlive.org/records/154gm-kvq48

**File format**: geoTIFF (.tif), arc ascii (.asc), ESRI shapefile (.shp), Keyhole Markup (.kml), and JavaScript Object Notation (.geojson).

**File naming convention:** MERIT_plus_05min_v2.2_{*Variable*}.{*Format*}

Where {*Variable*} is one of: IDs, IDsEnR, *flwdir, flwdirEnR or upstrArea*.

Where {*Format*} is one of: tif, asc, shp, kml, or geojson.

**Date Produced**: Dec 2023.

**Spatial Metadata**:

Extent: X: −180 to + 180

Extent Y: −60 to + 85 Resolution: 0.083333 decimal degrees (5 arc minutes)

Coordinate reference system: longitude/latitude reference system: longitude/latitude (WGS84 datum)

Projection in PROJ.4 notation: “ + proj = longlat + datum = WGS84”

Rows and columns: 1740, 4320

**Units:**

IDs: None

IDsEnR: None

flwdir: None

flwdirEnR: None

upstrArea: km^2^

**Nodata value**: Oceans, open-water, and Antarctica in the geoTIFF and ascii files have the no-data value of −9999. Exception, MERIT_plus_05min_v2.2_flwdir.tif has a nodata value of 247.

**File format**: tab delimited text (.csv)

**File naming convention:** MERIT_plus_05min_v2.2_{*Variable*}.csv Where {*Variable*} is one of: IDs or IDsEnR

**Units:**

IDs: None

IDsEnR: None

**MERIT-Plus 15 minute v2.2 Data:**

**Repository**: MSD-LIVE^33^, Project: Program on Coupled Human and Earth Systems (PCHES) https://data.msdlive.org/records/154gm-kvq48

**File format**: geoTIFF (.tif), arc ascii (.asc), ESRI shapefile (.shp), Keyhole Markup Language (.kml), and JavaScript Object Notation (.geojson).

**File naming convention:** MERIT_plus_15min_v2.2_{*Variable*}.{*Format*}

Where {*Variable*} is one of: IDs, IDsEnR, *flwdir, flwdirEnR or upstrArea*.

Where {*Format*} is one of: tif, asc, shp, kml, or geojson.

**Date Produced**: Dec 2023.

**Spatial Metadata**:

Extent: X: −180 to + 180

Extent Y: −60 to + 85 Resolution: 0.25 decimal degrees (15 minutes)

Coordinate reference system: longitude/latitude reference system: longitude/latitude (WGS84 datum)

Projection in PROJ.4 notation: “ + proj = longlat + datum = WGS84”

rows and columns: 580, 1440

**Units:**

IDs: None

IDsEnR: None

flwdir: None

flwdirEnR: None

upstrArea: km^2^

**Nodata value**: Oceans, open-water, and Antarctica in the geoTIFF and ascii files have the no-data value of −9999. Exception, MERIT_plus_15min_v2.2_flwdir.tif, has a nodata value of 247.

**File format**: tab delimited text (.csv)

**File naming convention:** MERIT_plus_15min_v2.2_{*Variable*}.csv Where {*Variable*} is one of: IDs or IDsEnR

**Units:**

IDs: None

IDsEnR: None

## Technical Validation

### MERIT-Plus river network datasets in 5 arc minute resolution

We used a multi-purpose 43 river network processing utility “networkTools” developed at UNH (https://github.com/wsag/WBM/tree/main/utilities) to build MERIT-Plus products by performing both identification and merging procedures with processing parameter values listed in Table 2. Network basin counts and other statistics are presented in Table 3.

**Table 3:** Summary of IHU MERIT and MERIT-Plus 5 arc minute network.

	Original by Eilander^6^	Identification	After Merging
Endorheic Basins (count)	Not identified	7,738	1,708
Exorheic Basins (count)	Not identified	122,966	122,966
Total Basins (count)	130,704	130,704	124,674
% Endorheic Area*	Not identified	19.17%	18.81%
False Endorheic Basins (count)	Not identified	12	0

*Excludes Antarctica.

The original IHU upscaled MERIT data set has 130,704 unique drainage basins all of which have outflow direction at their outlets equal to zero, and so cannot be identified as endorheic or exorheic. Most of these are small basins (91.2% of them are smaller than 10 grid cells in size or approximately 70 km^2^) that are located near ocean coastline and islands. Those numerous small coastal basins were found to be connected to the ocean through sub-pixel passages (e.g., fiords) resolved with a 10 m resolution land coastline dataset (see “Methods” section above) and composing chained coastal river outlet clusters (Fig. 4). Identification of endorheic basins yields a count of 7,738 basins that comprise 19% of the global land area (Table 3). Merging of the small endorheic basins that match filtering criteria (Table 2) reduces their count significantly to 1,708 while leaving the global endorheic land area almost unchanged. Visual checks of the merged basins indicate that most of them are in arid or semi-arid regions where formation of dunes or other landforms that block outlet passages are common.

In order to evaluate the procedures outlined above we used the UNH Water Balance Model (WBM^32^) to explore endorheic lake water accumulation. The question was whether any given endorheic basin would be likely to fill and overflow or will evapotranspiration rates exceed water accumulation rates, with the basin remaining hydrologically disconnected at the surface. The model was driven using 40 years of the MERRA2 historical climate drivers^36^ with all human hydrological components turned off^37^. WBM calculated endorheic lake water storage change using the difference between water inflow and evaporation from the lake surface where the latter is a function of storage and lake geometry and bathymetry. If the lake storage and size exceeds the depression capacity, then it is flagged as a false endorheic basin under historical climate conditions. Checking all endorheic basins by this criterion we found only 12 such outliers before merging, and after the merging none of those were found (Table 3).

**Fig. 6:**
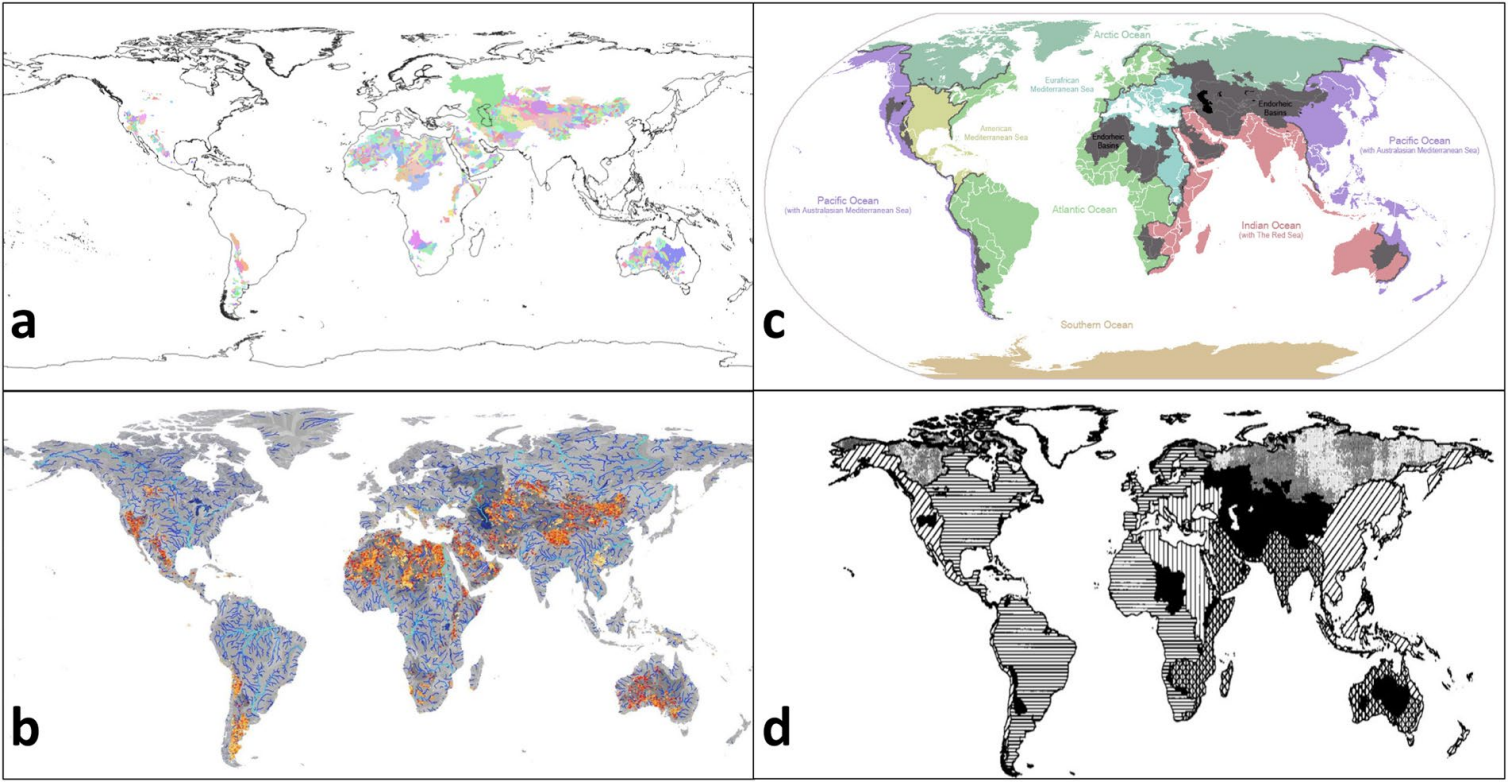
Comparison of endorheic regions from multiple sources. (**a**) MERIT-Plus dataset with endorheic basins shown in colours. (**b**) MERIT Hydro^5^ with endorheic basins shown in oranges and yellows and exorheic basins in blues and greys. (**c**) USGS Hydro1k^38^, with endorheic basins in grey and exorheic basins in colours. Source: Wikimedia.org. (**d**) STN-30p^1^ endorheic basins shown in black with exorheic basins as hatched lines.

We also checked the total/global endorheic land area and its location throughout the global land surface to the original MERIT Hydro^5^ and other known sources^1,5,38^ (Fig. 6). The match of MERIT-Plus to those is very good except the MERIT Hydro has a few extra locations in SW China, SE coast of South America, and few smaller areas in a wet temperate and tropical climate zone such as Indonesia. The authors of the latter dataset^5^ explain those as being karst drainage basins that are connected to the adjacent exorheic basins through the underground passages noting that those do not meet the common definition of “endorheic” basins by the connectivity to the ocean and, thus, are intrinsic to this particular dataset. Since we use basin connectivity to the ocean in the MERIT-Plus data, those (karst) endorheic area mismatches should be considered as an invalid basin type identification. The endorheic basin land fraction (18.81%, Table 3) match well with data from other sources^1,8,39^.

### MERIT-Plus river network datasets in 15 arc minute resolution

Production of the MERIT-Plus river network data in 15 arc minute resolution was created using the same approach and software with altered input parameters (Table 4) to adjust for the coarser grid cell size. For example, the “Maximum area” parameter (P1) was increased to 3000 km^2^ to account for extra space of inscribing of actual basin boundaries to the coarser grid.

**Table 4:** Parameters and their values used for MERIT-Plus data production in 15 arc minute resolution.

Parameter	Value	Comment
Identification		
Coastal segmentation	True	
Ocean mask	Mask file	Data from OpenStreetMaps
Ocean coast buffer	50 km	About double cell size
Merging (see Table 1)		
Maximum area (P1)	3000 km^2^	Visual equivalent of 60 × 50 km square
Maximum elevation delta (P2)	100 m	
Maximum flow path (P3)	0 km	Outlet is on the endorheic basin boundary
Pour point (Op1 & 2)	Op1 = False	The shortest distance from outlet to boundary
	Op2 = True	
Connecting (Op3 & 4)	Op3 = False	The highest upstream area in adjacent basin cells adjacent to the pour point cell
	Op4 = True	

Summary and statistics of the resulting MERIT-Plus endorheic basins at 15 minute resolution (Table 5) have approximately three times fewer basins as compared to the 5 minute network. However, the difference between endorheic basin numbers after identification and especially after merging are not that different since most of the small erroneous endorheic basins have been eliminated by the IHU upscaling process^6^.

**Table 5:** Summary of IHU MERIT and MERIT-Plus 15 min network.

	Original Eilander^6^	Identification	After Merging
Endorheic Basins (count)	Not identified	4,458	1,240
Exorheic Basins (count)	Not identified	31,933	31,933
Total (count)	36,391	36,391	33,173
% Endorheic Area	Not identified	18.07%	17.58%
False Endorheic Basins (count)	Not identified	2	0

*Excludes Antarctica.

Validation of the basin type by WBM endorheic lake simulation using the same logic and approach as for the 5 minute network found two false basins (Table 5) which were merged to adjacent exorheic basins.

The global distribution of 15 minute endorheic basins (Fig. 6) and their land fraction (Table 5) is similar to those of the 5 minute resolution network with the expected reduced granularity due to the coarser resolution.

### Uncertainty analysis

There are two aspects of the MERIT-Plus data production for the identification of endorheic and exorheic basins. The first one is relevant to assigning a Boolean value or flag for the basin endo- exorheic attribute without changing the flow direction data of the original source dataset^6^, and the second one is for merging small endorheic basins to host catchments. For both, we have conducted sensitivity analysis of our methods by the permutation of processing parameters that affect the output product. The uncertainty then is assessed from the variability of total endorheic area and match to known alternative endorheic basin maps described earlier in this section.

The summary of this analysis is given in the Table 6. The most important uncertainty analysis result is that ignoring or turning off the sub-grid cell ocean mask leads to significant mismatch of the resulting endorheic land areas to the known areas (indicated in the Table 6 “Location Mismatch” column) suggesting that the “observed” (this ocean mask) connectivity of basin outlets to the ocean is essential to produce the MERIT-Plus exo- and endorheic basin identification. Sensitivity, and, thus, the results uncertainty to the variability of the other dataset production parameters is fairly low assuring validation and quality of the data.

**Table 6:** Sensitivity analysis of the endorheic basin identification by the permutation of the key processing parameters in Table 2 used to produce the MERIT-Plus 5-min data.

Parameter	Value Change	Area change, %	Location Match
Ocean mask	On/Off	+0.00/+0.83	Yes/No
Ocean coast buffer, km	+5/−5	−0.02/+0.00	Yes/Yes
	+10/−10	−0.04/+2.57	Yes/No
Max area (P1), km^2^	+500/−500	−0.04/+0.05	Yes/Yes
	+1000/−1000	−0.07/+0.10	Yes/Yes
Other merging parameters	No changes since those do not affect area of endorheic basins		

## Data Availability

Code used in this paper is available here: https://github.com/wsag/WBM/tree/main/utilities. This GitHub wsag/WBM repository is licensed under the “GNU General Public License v3.0” and is one of the open-source public software access and use licenses. File names: 1. *networkTools*- Executable. Usage: » *networkTools -v JOB_PARAMETERS.init* 2. *networkTools_manual.init*- processing parameters *.init file template. Use corresponding options for the endorheic delineation of a given river network. Input/Output options and parameters are described in it.
